# Case report: Ponatinib as a bridge to CAR-T cells and subsequent maintenance in a patient with relapsed/refractory Philadelphia-like acute lymphoblastic leukemia

**DOI:** 10.3389/fonc.2022.1100105

**Published:** 2023-01-10

**Authors:** Fabio Giglio, Edoardo Campodonico, Francesca Lorentino, Maddalena Noviello, Elisabetta Xue, Raffaella Greco, Lorenzo Lazzari, Alessandro Bruno, Maria Teresa Lupo Stanghellini, Matteo Giovanni Carrabba, Roberta La Starza, Monica Casucci, Chiara Bonini, Sabina Chiaretti, Jacopo Peccatori, Robin Foà, Fabio Ciceri

**Affiliations:** ^1^ Hematology and Bone Marrow Transplantation Unit, IRCCS San Raffaele Scientific Institute, Milan, Italy; ^2^ Onco-hematology Unit, European Institute of Oncology, Milan, Italy; ^3^ School of Medicine, University Vita-Salute San Raffaele, Milan, Italy; ^4^ PhD Program in Public Health Department of Medicine and Surgery, University of Milano Bicocca, Milan, Italy; ^5^ Experimental Hematology Unit, San Raffaele Scientific Institute, Milan, Italy; ^6^ Cytogenetics and molecular medicine laboratory, department of Hematology and Clinical Immunology, University of Perugia, Perugia, Italy; ^7^ Innovative Immunotherapies Unit - Division of Immunology, Transplantation and Infectious Diseases - IRCCS San Raffaele Scientific Institute, Milan, Italy; ^8^ Hematology, Department of Translational and Precision Medicine, Sapienza University of Rome, Rome, Italy

**Keywords:** ponatinib, CAR-T, Philadelphia-like ALL, bridge therapy, maintenance therapy

## Abstract

Philadelphia (Ph)-like acute lymphoblastic leukemia (ALL) constitutes a heterogeneous subset of ALL with a uniformly unfavorable prognosis. The identification of mutations amenable to treatment with tyrosine kinase-inhibitors (TKIs) represents a promising field of investigation. We report the case of a young patient affected by relapsed/refractory Ph-like ALL treated with chimeric antigen receptor T (CAR-T) cells after successful bridging with compassionate-use ponatinib and low-dose prednisone. We restarted low-dose ponatinib maintenance three months later. Twenty months later, measurable residual disease negativity and B-cell aplasia persist. To the best of our knowledge, this is the first case reporting the use of ponatinib in Ph-like ALL as a bridge to and maintenance after CAR-T cell therapy.

## Introduction

Ph-like ALL, a subtype of B-cell ALL with adverse clinical features and unfavorable prognosis (1, 2), represents up to 15% of childhood ALL and 15-25% of adolescent and young adult ALL (3). They exhibit higher rates of measurable residual disease (MRD) persistence, higher rates of relapse, even in case of a MRD clearance, and a shorter survival compared to patients with non-Ph-like ALL (4). Patients with Ph-like ALL are often young males with hyperleukocytosis and a normal karyotype (5).

The pathophysiology of Ph-like ALL accounts for a plethora of kinase-activating mutations, affecting in up to 90% of cases either the tyrosine kinase super-family (*ABL1, ABL2, CSF1R* and *PDGFRB*) or the cytokine receptor pathway (*JAK1, JAK2, IL7-R, CRLF2, EPOR*), but lacking a classical *BCR::ABL1* rearrangement. Ph-like and Ph-positive ALL have a partly overlapping gene expression profile and are both often associated with deletions or mutations of the transcription factor *IKZF1* (6, 7).

The identification of actionable lesions in Ph-like ALL has paved the way towards targeted therapies (8). The efficacy of TKIs in Ph-like ALL has already been established (9, 10). In addition, other small molecule inhibitors, such as ruxolitinib, sirolimus and gedatolisib, have shown promising results in pre-clinical models of *JAK2*-mutated subtypes and are under evaluation (11). Moreover, the introduction of immunotherapy and CAR-T cells in the clinical practice may represent a valuable option to impact on the negative prognosis harboured by the Ph-like signature. As the treatment paradigm in ALL is undergoing a major shift, new efforts are warranted to define the proper place for each drug within the therapeutic algorithm for different subgroups of patients.

## Case presentation

We report the case of a 19-year-old female diagnosed with Ph-negative B-cell ALL in February 2019, who presented with hyperleukocytosis (WBC count 317 x 10^9^/L). Flow cytometry on a bone marrow (BM) aspirate showed that 93% of cells were positive for CD45, CD10, CD19 and CD22, and had an aberrant expression of CD33. FISH performed using the Cytocel probe detected a deletion at 6q21/SEC63 in 43.5% of the analysed nuclei. The *BCR::ABL1*-like predictor was positive and showed a *CRLF2* upregulation and an *IKZF1* deletion (7). Molecular-cytogenetic analysis, performed using the ZytoLight^®^ SPEC *CRLF2* Dual Color Break Apart probe, and the LSI *IGH* Dual Color, Break Apart Rearrangement Probe (Vysis-Abbott) showed hybridization patterns consistent with the presence of an *IGH : CRLF2* rearrangement. Targeted RNA sequencing detected no mutations or rearrangements.

The patient was enrolled in the chemo-immunotherapy GIMEMA LAL2317 protocol, which exploits a risk-oriented strategy based on disease characteristics and MRD evaluation at fixed time-points, intercalating a maximum of two cycles of blinatumomab into a pediatric-like chemotherapy backbone (clinicaltrial.gov NCT03367299).

Our patient, classified as very high risk, underwent three cycles of chemotherapy, obtaining a complete morphologic remission (CR) after induction. Central nervous system (CNS) prophylaxis was carried out as per protocol. MRD assessment by RQ-PCR Ig gene rearrangement remained strongly positive (>10^-2^) after all three chemotherapy cycles. After a single cycle of blinatumomab which induced the molecular remission (<10^-5^), the patient underwent an allogeneic hematopoietic stem cell transplant (HSCT) from an HLA-identical sister. Conditioning consisted of treosulfan, fludarabine and TBI 4 Gy, and the graft versus host disease (GvHD) prophylaxis included post-transplantation cyclophosphamide and sirolimus (12). A post-transplant aspirate documented a CR with full donor chimerism, FISH and a molecular MRD negativity. Sirolimus was discontinued six months later. The patient never developed GvHD.

In January 2021, 18 months after the HSCT, a BM evaluation detected a relapse (5% blasts). The patient had also a palpable mass in her right breast, whose histology was compatible with an ALL localization. No CNS disease was detected.

**Figure 1:**
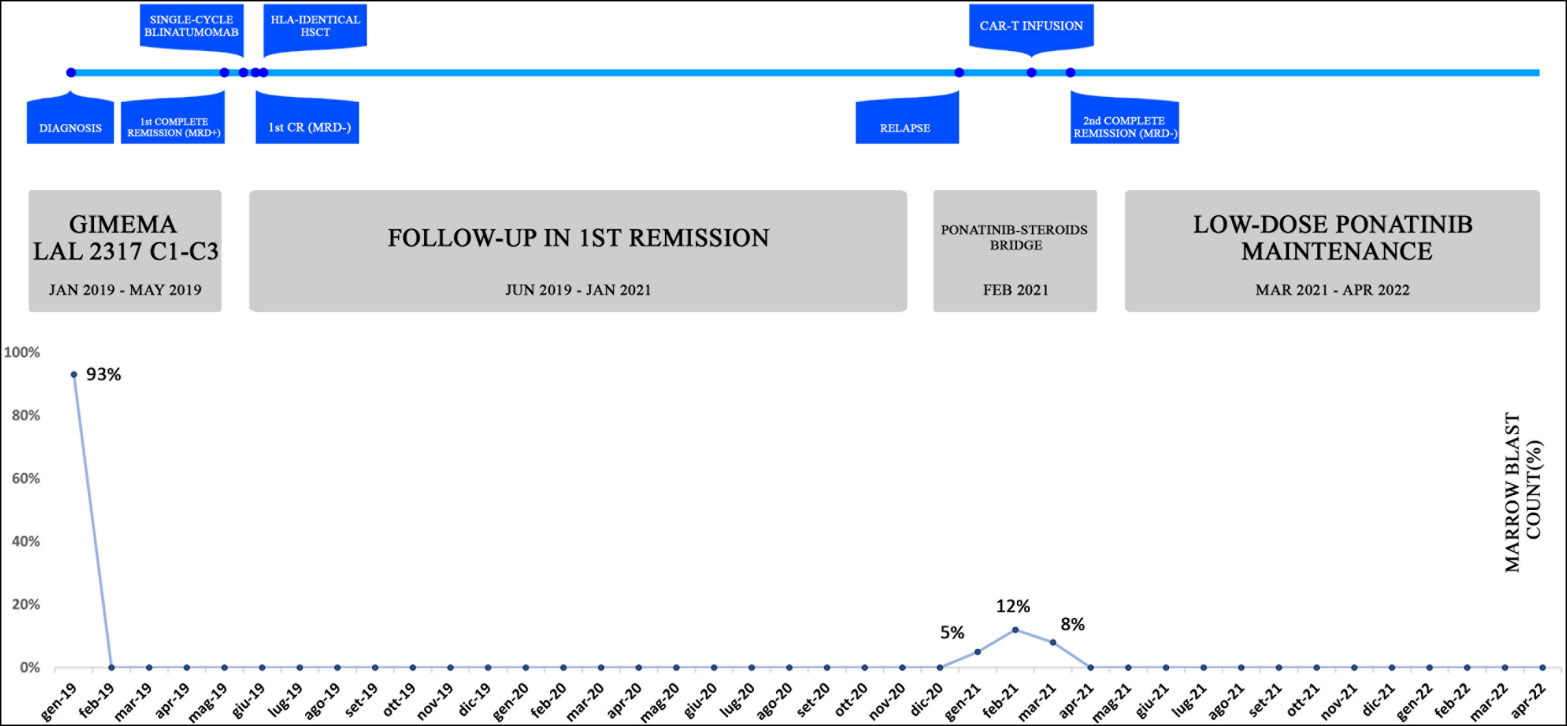
Case report timeline. The table offers an overview of the patient’s history, treatment lines **(A)** and corresponding marrow blast count **(B)**. The percentage of bone marrow blasts was evaluated at significant time-points, namely at diagnosis, post-induction, post-transplant, at disease relapse, during bridging and after CAR-T infusion. At the last follow-up, the patient is still in molecular remission and in persistent B-cell aplasia.

**Figure 2:**
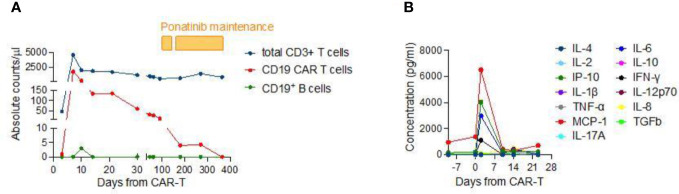
**(A)** Longitudinal evaluation of total CD3^+^ T cells, anti-CD19 CAR-T cells, CD19^+^ B cells and released cytokines in the patient’s peripheral blood. Absolute counts were evaluated by flow cytometry at several time-points for up to 1 year after CAR-T cell infusion. The pharmacokinetics shows a remarkable CAR-T cell expansion in the first week following infusion (coinciding with CRS onset and resolution), with engineered cells representing nearly 40% of overall T cells, and a subsequent drop in CAR-T cells over time. At day 270, the patient still has a subset of CAR-T cells accounting for around 1% of the total T-cell count. At the time of last follow-up, circulating CAR-T cells had decreased below the detection limit of the assay but B-cell aplasia persists. **(B)** Evaluation of serum cytokines/chemokines concentrations in the first three weeks after CAR-T cell infusion. The analysis shows a significant peak occurring within the first week after treatment.

The patient was deemed fit for anti-CD19 CAR-T cell therapy with tisagenlecleucel. After lymphapheresis in early February, we started a bridging treatment with ponatinib 45 mg daily for 30 days on compassionate use and 1 mg/kg prednisone for 14 days. No cardiac, hepatic or hematologic toxicity was reported. In mid-February, we repeated a BM aspirate that confirmed a morphologic relapse (12% blasts). A third aspirate after a month of ponatinib showed a stable disease (8% blasts) and on physical examination a reduction of the palpable breast nodule was documented. We withdrew bridging drugs and started lymphodepletion with fludarabine-cyclophosphamide, followed by a CAR-T cell infusion on March 2021. The patient developed a grade 4 neutropenia and received three doses of tocilizumab for grade 1 cytokine release syndrome (CRS). No neurotoxicity occurred. Three months later the patient was in CR with a full donor chimerism and a MRD negativity. A breast ultrasound revealed a regression of her nodule. In June 2021, maintenance with ponatinib at a lower dose (15 mg/day) was initiated. In November 2022, 20 months after the CAR-T cell infusion the patient is in good clinical conditions and in persistent molecular CR. She still receives ponatinib maintenance with excellent tolerance, except for a 10-day discontinuation due to a transient G4 neutropenia. **Figure 1** summarizes the case timeline. We longitudinally monitored the patient’s CAR-T cell expansion and their subsequent persistence by flow cytometry and plotted the data over time in **Figure 2A**. After a marked expansion peak at day 7 after infusion (1819.6/mcl), CAR-T cell counts decreased, though persisting over time. At late time-points (day 180 and 270), the patient still had circulating anti-CD19 CAR-T cells (4.0 and 4.3/mcl, respectively). At the last available follow-up (365 days, April 2022), circulating CAR-T cells were no longer detectable. However, a concomitant B-cell aplasia is ongoing and we are monitoring it as a decisional tool for future treatment. Peak levels of inflammatory cytokines and chemokines occurred within the first week after the CAR T-cell infusion, with particularly high levels of monocyte chemoattractant protein-1 (MCP-1), interferon γ-induced protein 10 kDa (IP-10), interleukin-6 (IL-6) and interferon-γ (IFN- γ, **Figure 2B**).

## Discussion

The present case illustrates how ponatinib might represent a valid therapeutic option to be explored in Ph-like ALL. Even though TKIs have to date no standardized place, it seems reasonable to incorporate them in treatment schemes given their safety and potential effectiveness. The patient was initially enrolled into a sequential chemo-immunotherapy protocol (clinicaltrial.gov NCT03367299) and obtained a MRD negativity only after a cycle of blinatumomab, suggesting a possible role of this drug in Ph-like patients, whose long-term efficacy is still debatable.

Upon relapse, anti-CD19 CAR-T cells were considered the best salvage option. Considering the fast disease kinetics, the risk of major complications while waiting for the CAR-T cells and the widely accepted notion that a lower disease burden upon lymphodepletion correlates with an improved outcome, it seemed imperative to choose a safe and effective bridging option. Based on the assumption that *CRLF2* hyperexpression might be amenable to treatment with ponatinib, our patient received 45 mg ponatinib daily for a month and steroids for two weeks: follow-up BM aspirate before lymphodepletion demonstrated a persistent disease stability in spite of the rapidly progressive nature of Ph-like ALL. Even though it is difficult to discriminate between the role of ponatinib and the role of steroids due to their synergistic effect, the combination proved effective.

Whereas the efficacy of TKIs in *ABL1*-mutated ALL is demonstrated by a growing number of studies (13), there is still uncertainty on to their role in cases lacking such mutations, the rationale of its efficacy lying in the broad-spectrum of its kinase-inhibiting activity. Interestingly, ponatinib might represent the most promising of all TKIs based on studies highlighting its efficacy regardless of the patient’s mutational status, both *in vitro* and *in vivo* (7, 14). Recently, *Lunghi et al.* (15) reported a patient with relapsed/refractory Ph-like ALL with *BCR::JAK2* rearrangement who achieved a CR2 and a first MRD clearance with ponatinib.

Another open issue is how to consolidate and maintain the results obtained with CAR-T cells. Even though HSCT consolidation seems beneficial in specific cases, clear indications are missing. Due to the major toxicities and the poor outcome associated with a second HSCT, we decided to strictly monitor the MRD status and ongoing B-cell aplasia, while pursuing a maintenance therapy with lower-dose ponatinib. Pre-clinical data show that TKIs might affect T-cell receptor signaling. It is already established that dasatinib inhibits Src family kinase activity, potentially affecting the effectiveness of immunotherapies. More recently, it has been reported that dasatinib may also ablate CAR-mediated signaling, by interfering with LCK and inhibiting the phosphorylation of CD3z and ZAP70 (16). This activity can induce a reversible function-off state in CAR-T cells that can be exploited to mitigate CRS (16) and to improve CAR-T cell fitness by preventing exhaustion and promoting the acquisition of a memory-like phenotype (17). However, little is known about ponatinib immunomodulating properties. Small clinical series suggest that coadministration of ponatinib or dasatinib with immunotherapies do not affect their effectiveness and might be beneficial in disease control (18).

To the best of our knowledge, this is one of the first reports of successful treatment of non *ABL*-mutated Ph-like ALL with ponatinib and the very first report of ponatinib being used as a bridge to and maintenance after CAR-T cell therapy.

The current treatment landscape in ALL is rapidly evolving. Unfortunately, some subsets of ALL are lagging behind and still retain a poor prognosis. Several open issues require settling in Ph-like ALL, such as the role of TKIs, which inhibitor to prefer, the appropriate timing of its introduction, and the outcomes of combination therapies. There is also an urgent need to define a standardized bridging strategy to CAR-T cells and post-CAR-T cell management in ALL. Future studies, preferably prospective and randomized, are warranted in order to re-define the appropriate therapeutic algorithm for patients with Ph-like ALL.

## Data availability statement

The datasets presented in this article are not readily available because of ethical/privacy restrictions. Requests to access the datasets should be directed to the corresponding author.

## Ethics statement

The studies involving human participants were reviewed and approved by the patient has been treated according to current institutional guidelines, upon written informed consent for all the treatment procedures, the review of medical records and the use of immunological monitoring for patients undergoing allogeneic HSCT within the non-interventional “ALMON study”, approved by San Raffaele Institutional Ethical Committee on 19/10/2007. Bone marrow and peripheral blood samples were collected and stocked at San Raffaele Hospital upon written informed consent for future potential studies within our Institutional observational “biobanking protocol”, approved by San Raffaele Institutional Ethical Committee on 04/05/2006.The patients/participants provided their written informed consent to participate in this study. Written informed consent was obtained from the individual(s) for the publication of any potentially identifiable images or data included in this article.

## Author contributions

FG conceived the study and wrote the paper; EC wrote the paper and provided all clinical and hematological data; FL, EX, RG, LL, AB, ML-S, MGC, JP, FC provided all clinical and hematological data; MN and CB performed and evaluated CAR-T cell monitoring; RS performed and evaluated cytogenetic analysis; MC performed and evaluated serum cytokines/chemokines concentrations; SC evaluated and performed the BCR/ABL1-like predictor. RLS, SC, RF, FC supervised and wrote the paper. All authors contributed to the article and approved the submitted version.
